# Automated facial characterization and image retrieval by convolutional neural networks

**DOI:** 10.3389/frai.2023.1230383

**Published:** 2023-12-20

**Authors:** Syed Taimoor Hussain Shah, Syed Adil Hussain Shah, Shahzad Ahmad Qureshi, Angelo Di Terlizzi, Marco Agostino Deriu

**Affiliations:** ^1^PolitoBIOMed Lab, Department of Mechanical and Aerospace Engineering, Politecnico di Torino, Turin, Italy; ^2^Department of Research and Development (R&D), GPI SpA, Trento, Italy; ^3^Department of Computer and Information Sciences, Pakistan Institute of Engineering and Applied Sciences, Islamabad, Pakistan

**Keywords:** oriented gradient-based algorithm, convolutional neural networks, GoogLeNet, AlexNet, KNN, computer vision, facial features extraction

## Abstract

**Introduction:**

Developing efficient methods to infer relations among different faces consisting of numerous expressions or on the same face at different times (e.g., disease progression) is an open issue in imaging related research. In this study, we present a novel method for facial feature extraction, characterization, and identification based on classical computer vision coupled with deep learning and, more specifically, convolutional neural networks.

**Methods:**

We describe the hybrid face characterization system named FRetrAIval (FRAI), which is a hybrid of the GoogleNet and the AlexNet Neural Network (NN) models. Images analyzed by the FRAI network are preprocessed by computer vision techniques such as the oriented gradient-based algorithm that can extract only the face region from any kind of picture. The Aligned Face dataset (AFD) was used to train and test the FRAI solution for extracting image features. The Labeled Faces in the Wild (LFW) holdout dataset has been used for external validation.

**Results and discussion:**

Overall, in comparison to previous techniques, our methodology has shown much better results on k-Nearest Neighbors (KNN) by yielding the maximum precision, recall, F1, and F2 score values (92.00, 92.66, 92.33, and 92.52%, respectively) for AFD and (95.00% for each variable) for LFW dataset, which were used as training and testing datasets. The FRAI model may be potentially used in healthcare and criminology as well as many other applications where it is important to quickly identify face features such as fingerprint for a specific identification target.

## 1 Introduction

The current era is representing the correct essence of a proverb said by a Chinese philosopher, “A picture is worth a thousand words” (Huffer et al., 2019). Images have an important role in visual-based information since a picture can communicate very complex ideas in a relatively simple manner. However, processing and handling large data are cumbersome tasks. Since information retrieved is useless if the essence of the required information is missing in the output (Yang et al., 2008), efficient and minimal time response systems are required.

With the advancement of multimedia-based technology, different firms came to the frontline. They provided different platforms like Facebook, Twitter, WhatsApp, Amazon and eBay (Zhang and Wang, 2012). Each platform is itself a big ocean for multimedia data. On this huge data, different recommendation systems are working together. They employ valuable information and recommendations to the end-users according to their needs (Fayyaz et al., 2020). Similarly, these platforms led the researchers to develop more better and efficient information retrieval algorithms.

Content-Based Image Retrieval (CBIR) is a hot research field which is getting the attention of researchers to fill the needs of the current era. The used features define the success of CBIR (Raghuwanshi and Tyagi, 2019). Initially, researchers mainly focused on techniques for data retrieval by text-based query within minimal time and accuracy (Chang and Fu, 1980a,b; Chang and Kunii, 1981; Tamura and Yokoya, 1984; Chang et al., 1988, 1998; Chang and Hsu, 1992). However, it was challenging to annotate and manage the large data. Then researchers felt the need for new efficient ways for the retrieval of image-based data (Tyagi, 2017). Through integrated efforts, a new technique was introduced in the form of features extraction like shape (Mezaris et al., 2004; Zhang and Lu, 2004; Yang et al., 2005, 2008; Zhang et al., 2009) based information and color-based features (Flickner et al., 1995; Pass and Pass, 1996; Park et al., 2013).

Since the past few years, several researchers have been contributing to facial-based feature extraction and recognition (Samal and Iyengar, 1992; Brunelli and Poggio, 1993; Valentin et al., 1994; Chellappa et al., 1995). In general, face recognition systems are developed with two steps: (1) face detection and (2) face recognition. For face detection, a number of different methods have been introduced, such as edge representation (Jesorsky et al., 2001; Singh et al., 2016), gray information (Maio and Maltoni, 2000; Feng and Yuen, 2001), color-based information (Dai and Nakano, 1996), neural network-based detection (Li et al., 2015), morphology-based preprocessing (Han et al., 2000), and geometrical face model (Jeng et al., 1998). However, for face recognition, the following different methods have been proposed: Eigenfaces (Swets, 1996; Zhang et al., 1997), hidden Markov model (Nefian and Hayes, 1998), LDA based techniques (Swets, 1996; Belhumeur et al., 1997), using local autocorrelations and multiscale integration (Goudail et al., 1996), discriminant eigenfeatures (Swets, 1996), algebraic feature extraction method (Liu et al., 1993), and probabilistic visual learning for object representation (Moghaddam and Pentland, 1997). In parallel to facial recognition, researchers are also paying attention and progressing toward facial expressions that may be applied in different fields such as pain intensity determination (Othman et al., 2023), health prediction (Yiew et al., 2023), vehicle driving (Ashlin Deepa et al., 2023), security checking (Mao et al., 2023), facial expressions of babies to predict their health status (Brahnam et al., 2023), and prediction of different diseases such as neurological outcome and pain. In this regard, Jiang and Yin (2022) developed a convolutional attention-based facial expression recognition system with a multi-feature fusion method. These authors focused on the convolutional attention-based module that learned facial expressions in a better way. In another study, Mao et al. (2023) used a customized convolutional deep learning model to train facial expressions. Their main focus was on deploying an region of interest (ROI) pooling layer with L2 regularization, including learning rate decay mechanisms to train well on facial expression landmarks.

In facial recognition, Mahmood et al. (2022) suggested a facial image retrieval technique that deals with image texture features with defined pixel patterns such as local binary, ternary, and tetra directional pixel patterns of the input image. The PCA optimizer function was used to select the robust features from a collection of extractive feature sets. Finally, these authors used the Manhattan distance equation to calculate feature similarity.

Zhang et al. (2021) proposed a novel data structural technique named as the deep hashing method that focuses on the improvement of image retrieval speed with low memory consumption. In addition, on the basis of deep feature similarity, Zhang's model generates the hashing code of the features of the fully connected layer from convolutional neural network (CNN) and develops a hashing database for a retrieval purpose. In this database, the associated label of the image generates a label matrix for the classification and retrieval of performance purposes. Then, the hamming cube learning technique has been used to compute the central image features of the inter-class separability that assisted in the retrieval process against the query image.

Sato et al. (2019) presented a peculiar face retrieval framework. In a situation, a user wants to find a particular person with one visual memory about that person. The user selects some pictures about that person based on target specific information and passes them to the trained model. Deep convolutional network processes and the resulting features are then matched for retrieval. Hasnat et al. (2019) proposed a new efficient method of face image retrieval system, i.e., discriminative ternary census transforms histogram (DTCTH) technique, which is specialized for capturing only the required information. Later, Shukla and Kanungo (2019) also introduced a new approach for face recognition and retrieval, in which a bag of features is used by extracting visual words with the help of the Gray Wolf Optimization algorithm. Wu et al. (2010) proposed a new method for face-based image retrieval using indexing. They extracted local and global features of faces and quantized the local features containing the special properties of faces into visual elements. Sun et al. (2019) introduced a new technique for facial images that show large variations in illumination, pose, and facial expressions. This technique combined the Face++ algorithm (Fan et al., 2014) and convolutional neural networks for image retrieval.

Tarawneh et al. (2019) used deep learning methods to retrieve images by analyzing the face mesh. In their study, the authors used VGG16 and AlexNet as the main focusing networks for training and testing. With the help of these networks, different feature representation approaches of the faces have been analyzed on different layers of the network and then used for the retrieval process against each query image.

In previous studies, different combinations of methods with convolutional neural networks have been reported for retrieval purposes. Some of these methods increase the complexity of the pipeline while others also require improvement toward a more efficient retrieval of the right information in a short time.

In this study, we present a novel pipeline for image retrieval that merges the power of two well-known tools, i.e., GoogleNet and AlexNet. In addition, we have also developed a novel convolutional neural network named as FRetrAIval (FRAI) for the training of face recognition.^1^ Two different standard datasets for training and testing purposes have been considered: AFD (training and validation) and LFW (Gary et al., 2008) (external validation), which have shown better classification and retrieval results in accuracy, recall, and precision measures.

## 2 Materials and methods

In this study, we used two different standard datasets named as aligned face dataset (AFD) (see text footnote 1) and LFW database (Gary et al., 2008). In both datasets, aside from only numerous facial characters for image retrieval purposes, they are having different facial expressions such as happy, sad, neutral, angry, and surprise, as shown in **Figure 10**. This aspect suggests that the model's recognition is based not only on facial landmarks but also recognizing expressions and appearances. In addition, AFD has a good number of samples per class as compared to the other holdout dataset, which consequently encouraged us to use this dataset for training on our proposed GoogLeNet CNN. After training, the LFW dataset was used for the testing phase of our methodology and is elaborated further in the Section 3.

### 2.1 Dataset and image preprocessing

A. In the AFD (see text footnote 1), over 10,000 images of 100 different celebrities were collected from Pinterest. An average of 100 images of each celebrity was included in this dataset.

**Figure 1:**
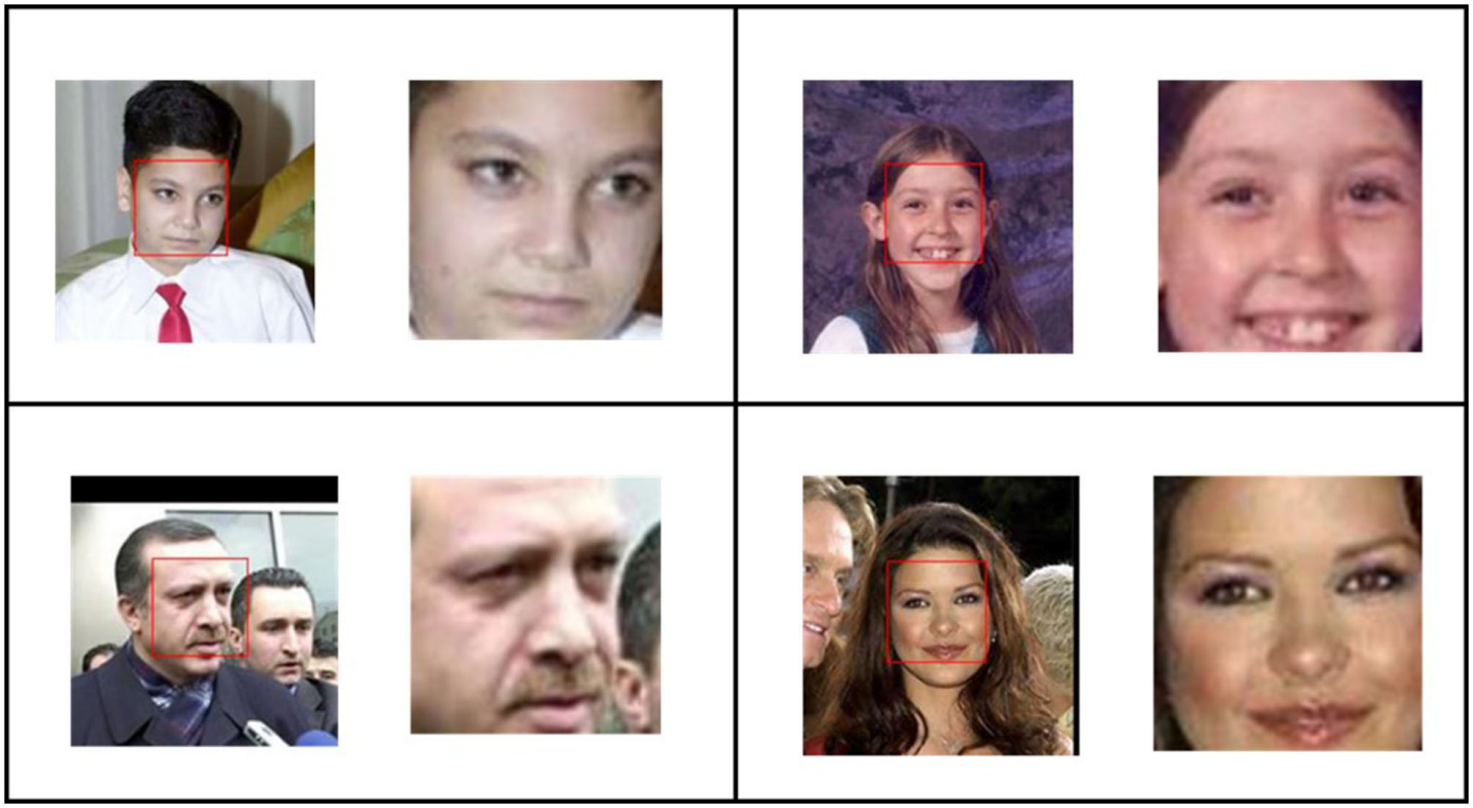
Face region selection using an oriented gradient-based algorithm (Dalal and Triggs, 2005) on AFW dataset faces; left image in each cell is original where red box is representing the detected face and right one is the automatically cropped face region.

B. A database based on unconstrained face recognition, i.e., LFW database (Gary et al., 2008), was used in this study. It comprises 13,233 faces with 5,749 unique people collected from the web. All images comprised the face of some characters and were labeled with the character's name. In this dataset, 1,680 images had two or more distinct photos. As shown in Figure 1, dataset images were preprocessed with the help of a local computer-based crawler (Dalal and Triggs, 2005).

**Figure 2:**
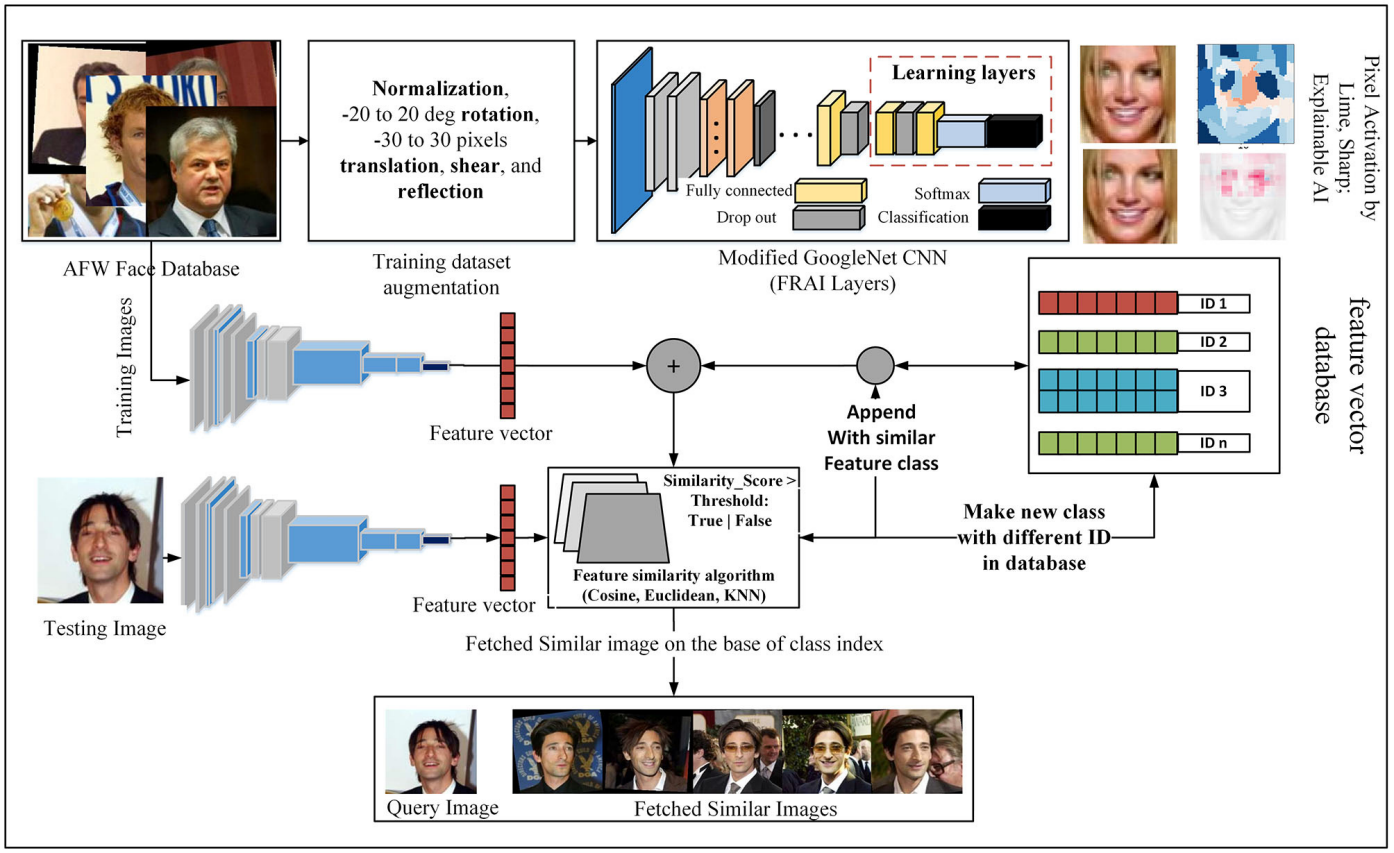
An end-to-end automatic facial based image retrieval system.

This program iteratively goes to the local disk drive and fetches an image. Image names were decoded into labels and stored in the absolute path in a database with the corresponding labels as shown in the flow diagram of our methodology in Figure 2. Each image of the database was fetched and passed on to the face detection model, which was based on a histogram of an oriented gradient-based algorithm (Dalal and Triggs, 2005). The face-selection region thus obtained was stored in the labeled face folders. Then, we loaded the data according to their labels and used the augmentation technique.

### 2.2 Integration of GoogleNet and AlexNet CNNs

GoogleNet (Szegedy et al., 2014) and AlexNet networks were the two CNNs considered for this study. GoogleNet (Szegedy et al., 2014) is a convolutional neural network having a total of 144 layers and comprising convolutional, pooling, fully connected, and softmax layers. In the AlexNet network, there is a total of nine layers, including an input layer and convolution and fully connected layers. As the last layers of this network, the AlexNet team introduced three fully connected layers.

**Figure 3:**
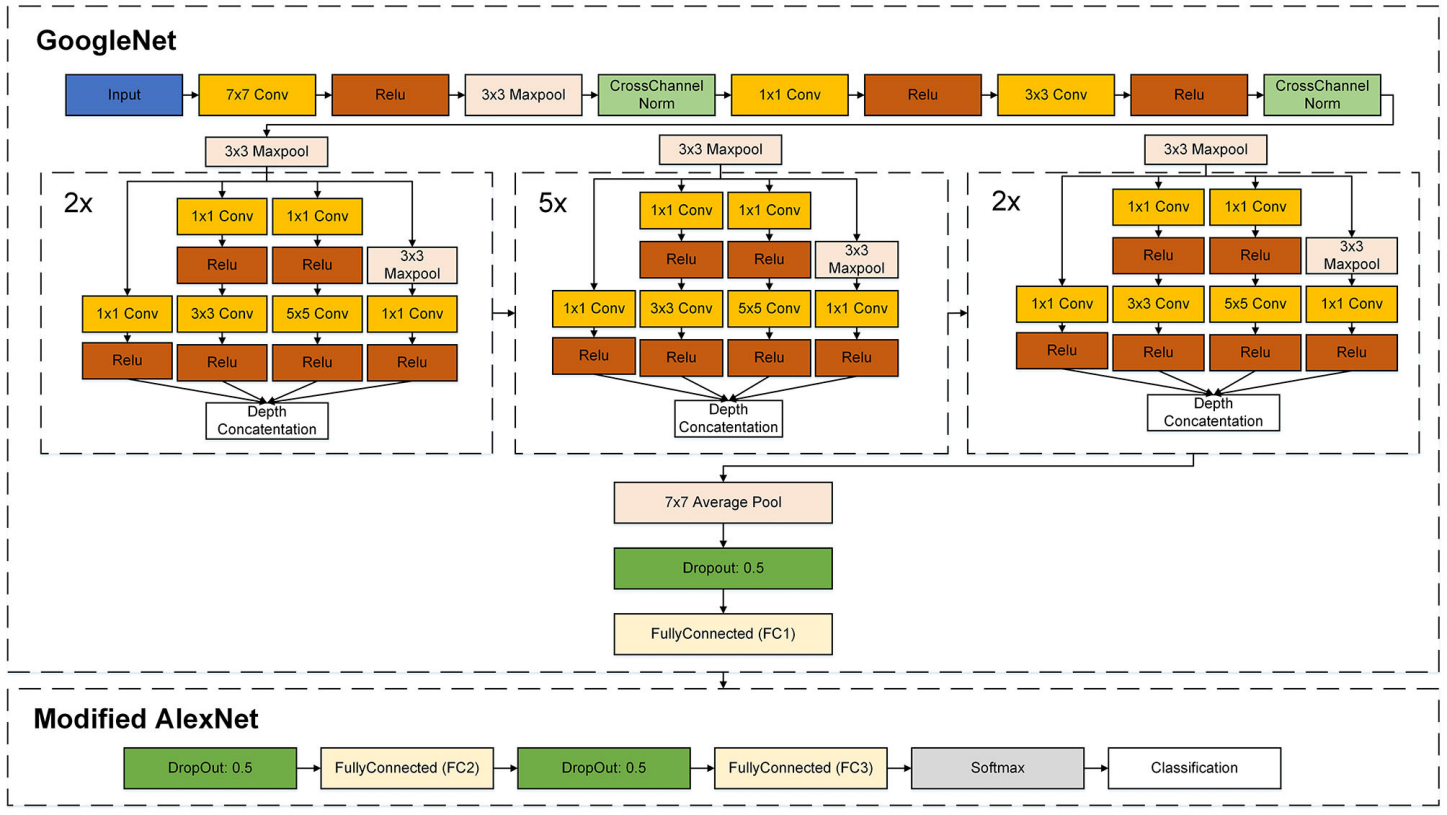
Summary of proposed novel FRAI layers.

The FRAI network uses a modified version of the GoogleNet network in which three further layers are added, as shown in Figure 3. Two of those layers were taken from the AlexNet (Krizhevsky et al., n.d.) network, of which the last two fully connected layers were made by 500 interconnected neurons. A final fully connected layer was added, i.e., the classification layer having the same number of neurons as the number of training dataset characters. In other words, the last classification layer is a set based on the required number of classes.

For the FRAI architecture, we chose these two networks due to their application in many research studies of image recognition tasks such as those in the studies of Wu et al. (2015) and Mehendale (2020). Individually, GoogleNet is efficient in parameter usage space such as inception behavior, which uses multiple filter sizes, such as 1 × 1, 3 × 3, and 5 × 5, within the same layer. This allows for the network to capture features at different spatial scales without significantly increasing the number of parameters. In addition, it uses the 1 × 1 filter size in the inception modules to reduce the computational complexity. Moreover, its inception module has inspired other CNN architectures. As a result, many other architectures have incorporated this module. On the other hand, we used the behavior of the last fully connected layer of AlexNet by using the same size fully connected with the alteration of dropout layers for better generalization. These visions led toward better generalization and robustness in the facial characterization of our model.

Moreover, in the FRAI network, each newly added fully connected layer (from AlexNet) was further connected to the dropout layer by having a 50% dropout probability. This strategy helps the system to pass the best information to the next layer from the previous one.

### 2.3 FRAI pipeline

In the section, a description of the FRAI pipeline has been discussed in detail, as shown in Figure 2. We started with dataset preprocessing followed by dataset grouping into training and testing sets. In this grouping, the FRAI model was trained on the training set. After training, the last classification layer was removed, and the model started to obtain well-represented features or face vectors for the development of a database. During the initial stage of database development, we computed a threshold (as is discussed for each retrieval algorithm in Section 3) using the hit-and-trial method to decide using the holdout LFW dataset images if they match too closely to a character that exists already in the database or does not exist. This technique helps in designing an automation pipeline to decide whether, for the current character, a new index should be introduced or not. After developing a good database, we started the retrieval process with the help of query images. With similar steps for the query process, the first query image features were computed and then passed to the feature matching algorithms. These algorithms took a decision based on the threshold of whether matching faces exist in the database or not. If a matching face does not exist, then the FRAI pipeline considers that person as a new one and adds their image with a new index. In this manner, our automated pipeline itself expands the database with new upcoming faces/characters.

### 2.4 Similarity metrics

In the present study, the following similarity metrics were used to compute the accuracy:

#### 2.4.1 Euclidean distance

In this matrix, the formula (Malkauthekar, 2013) was used.


(1) $$D_{euc}=\;\sqrt{\sum_{k=1}^{n}{\left(x_{k}-y_{k}\;\right)}^{2}},$$

where *x* is a query image vector, *y* is a database image vector, and *k* is representing the column number.

#### 2.4.2 Cosine similarity

In this similarity measure, we have used the formula (Lahitani et al., 2016) as


(2) $$D_{cosine}=\cos\left(\theta \right)=\frac{x\;.\;y}{\left|\left|y\right|\right|\;\left|\left|x\right|\right|}=\frac{\sum_{i=1}^{n}x_{i}y_{i}}{\sqrt{\sum_{i=1}^{n}x_{i}^{2}}\sqrt{\sum_{i=1}^{n}y_{i}^{2}}},$$

where *x* is query image vector and *y* is database image vector and *i* is representing the column number.

#### 2.4.3 k-NN algorithm

This algorithm (Guo et al., 2003; Moldagulova and Sulaiman, 2017) works with the value of k for the prediction of the label. When the label is predicted, then the accuracy is 100% but otherwise 0% because this algorithm returns only the best-suited class from the vicinity of the query vector.

In the present study, we used different metrics algorithms for getting better results than the previously proposed techniques. In this system, the vectors of an extracted query image features match those of the database features and indicate their similarity score. If the score is lower than the defined threshold, then it will be part of the database as a new feature. Afterward, images were stored in our database with its features for future use.

## 3 Results and discussion

Two different standard datasets, Aligned Face and LFW datasets, were used in this study, and these experimentations were carried using Dell Alienware machine, Intel (R) Core (TM) i7-−7700 HQ CPU @ 2.80 GHz, having 32 GB RAM. Initially, we selected the Aligned Face dataset for training because this dataset has a good number of samples per class as compared to the LFW dataset, and convolutional neural networks also require a good/huge number of samples for training (Asmat et al., 2021; Pande et al., 2022). After the selection of the dataset, we employed augmentation (Khalifa et al., 2021) to increase the number of samples further. In augmentation, we applied different parameters such as normalization, i.e., −20° to 20° for rotation and −30 to 30 pixels for translation and shear, and even reflection. With these augmented images, we started to train the FRAI. To reach the best training results, we employed the hit-and-trial method to find the best parameters for our convolutional neural network (CNN). These parameters were minibatch size, total iterations for training, and learning rate, which were optimized using Adam (Kingma and Ba, 2015), which is an optimization algorithm, and 104, 20, and 0.0003 were the optimized values of the minibatch size, total iterations for training, and learning rate parameters, respectively. At the outset of our study, we initiated the training of our FRAI network with carefully randomly selected parameters. As the training progressed, we observed that, after ~650 iterations, further improvements in validation accuracy were minimal, and this pattern continued until we reached a total of 1,640 iterations. To prevent overfitting of the training model, we retrained and stopped the training earlier than expected. The reason behind learning in fewer transactions was our efficient preprocessing of faces. The cropped characters' faces helped the model in focusing only in the close vicinity to learn the better convolutional features. The second point in this scenario is the batch size of 104 images. From these consecutive numbers of images, it is implied that the model learned in a few epochs. The last important point is that we used the weights of GoogleNet that helped in the training to bring less changes to weights in the GoogleNet layers. Adversely, the last newly introduced two fully connected layers altering pooling layers will be trained with more changing weights.

**Figure 4:**
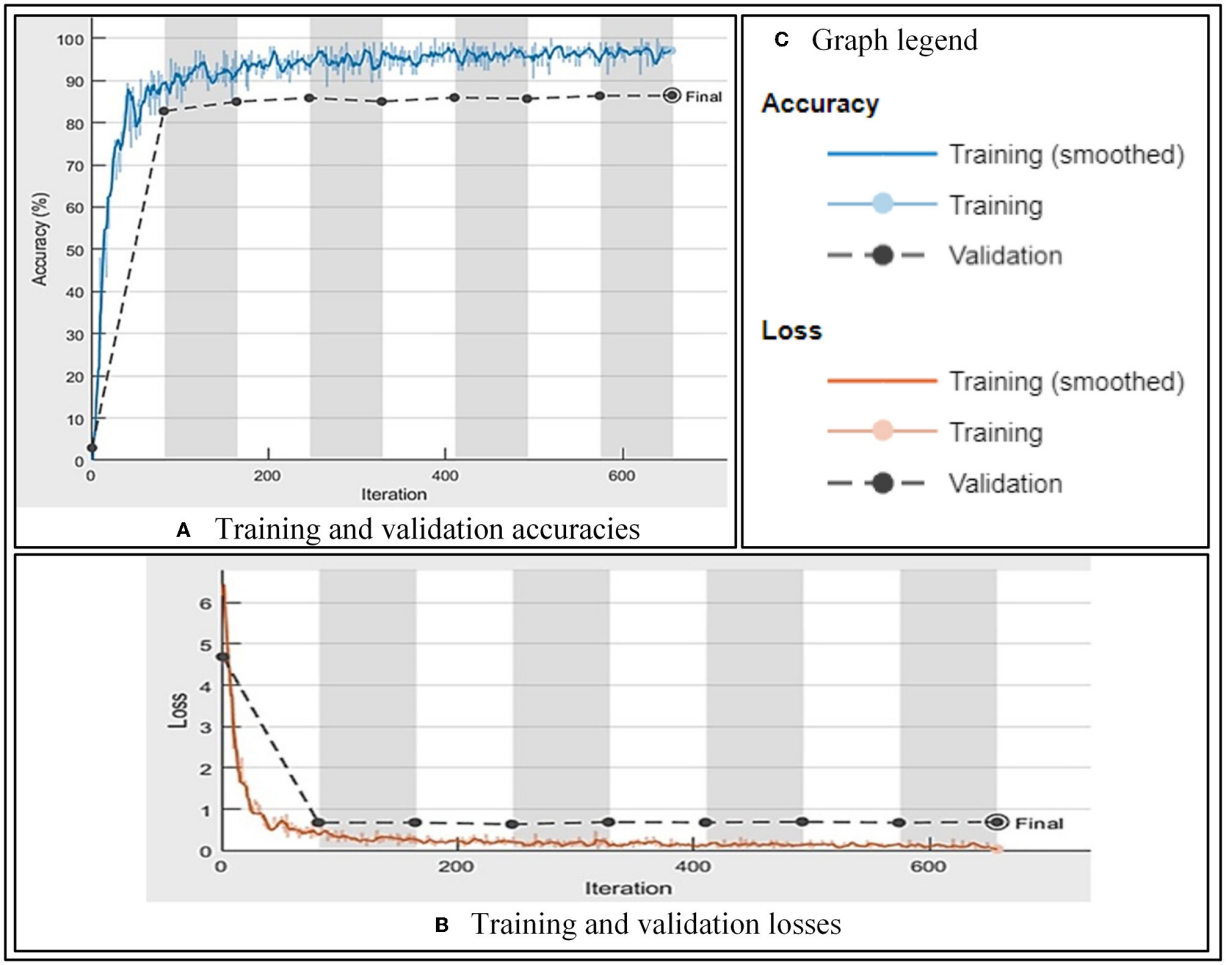
FRAI training and validation performance. **(A)** Training and validation accuracies. **(B)** Training and validation losses. **(C)** Graph legend.

On the best-found parameters, the CNN has shown a validation accuracy of 85.97% with 0.07% of training loss against 3:1:1 split in the dataset as training, validation, and testing. On testing, the FRAI trained model resulted in 82, 97.29, 83.16, 85.39, and 81.05% performance measures for accuracy, AUC, F-1, precision, and recall values, respectively. The mentioned training and validation results of our FRAI are shown in Figures 4A–C as training accuracy, training loss, and parameters, respectively. The training graph shows the learning behavior of the network with validation accuracy. The training accuracy resulted in a total of 656 iterations, after which the training was stopped, as also shown in Figure 4. Similarly, the training loss showed the opposite behavior to the training loss toward minimization with validation loss. After the successful completion of the training, we removed the last layer, i.e., softmax, and started to retrieve feature vectors.

To provide a more comprehensive understanding of the learning capabilities of the FRAI model, the ablation (layer-freezing) technique was used to compute the accuracy on the layers of the non-ablated (non-freeze) training model by deploying the same AFD dataset's training, validation, and testing sets. To facilitate comparison, the FRAI model's layers were divided into four quartiles: the first experiment covered layers up to 0.25, the second experiment covered layers up to 0.50, the third experiment covered layers up to 0.75, and the final experiment involved training with all layers, without ablation. These quartiles were applied in two fashions: in a forward way, the FRAI network was kept frozen from the start to the end, and in a second way, the FRAI model was kept frozen from the end to the start, such as transfer learning. In the forward way, we found that, up to the first quartile layers, the model yielded a 14% accuracy by training on ~38 million parameters. For the second experiment, up to second quartile layers, the model showed around 38% accuracy considering ~42 million parameters. For the third experiment, up to third quartile layers, the model resulted in 45% accuracy by training on ~46 million parameters, and at the end, the whole networks' training produced 82% accuracy with the training of ~47 million parameters. On the other hand, in second way from the end to the start, we found that, up to the first quartile layers, the model yielded into an accuracy of 79% by training on ~35 million parameters. For the second experiment, up to second quartile layers, the model showed ~75% accuracy considering ~30 million parameters. For the third experiment, up to third quartile layers, the model resulted in 70% accuracy by training on ~27 million parameters, and at the end, whole networks' training produced 82% accuracy with ~47 million parameters' training.

**Figure 5:**
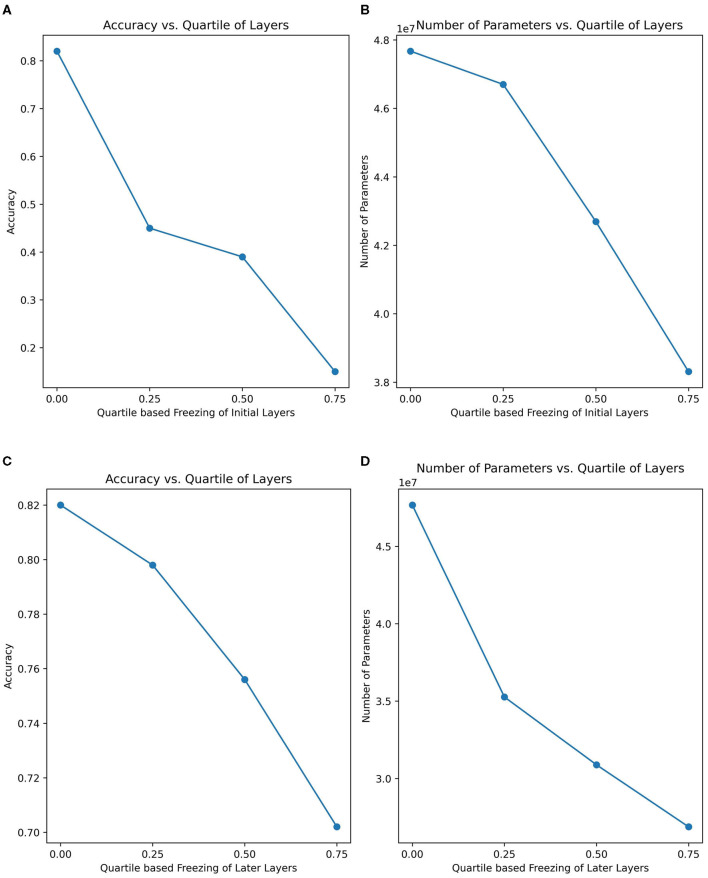
Comparison of training performance in ablation process. **(A)** Comparison between accuracy and quartile-based freezing of layers in forward way. **(B)** Comparison between number of parameters and quartile-based freezing of layers in forward way. **(C)** Comparison between accuracy and quartile-based freezing of layers in backward way. **(D)** Comparison between number of parameters and quartile-based freezing of layers in backward way.

This analysis highlighted the significance of training the entire model when considering the layers within the first quartile. Skipping the training of these layers led to decreased accuracy, but including their training significantly improved the overall accuracy. In the forward training approach, it was observed that, when the ending layers were considered for training, the model lacked the necessary weights to recognize facial landmarks accurately, which is crucial for achieving high accuracy in image recognition, particularly in scenarios involving diverse facial expressions and appearances, as depicted in Figure 5. In the general conclusion for ablation, we highlight the crucial insights that the original weights alone do not well-characterize the faces posing various complex human emotions. It became evident that employing pre-trained weights and training the entire model was essential. This approach allowed for the refinement of weights across all layers, facilitating the ideal combination of features. Specifically, the initial layers learned to discern edges, corners, and texture details, the middle layers integrated these elements to form shapes, and the later layers refined connections and made accurate shape predictions.

**Figure 6:**
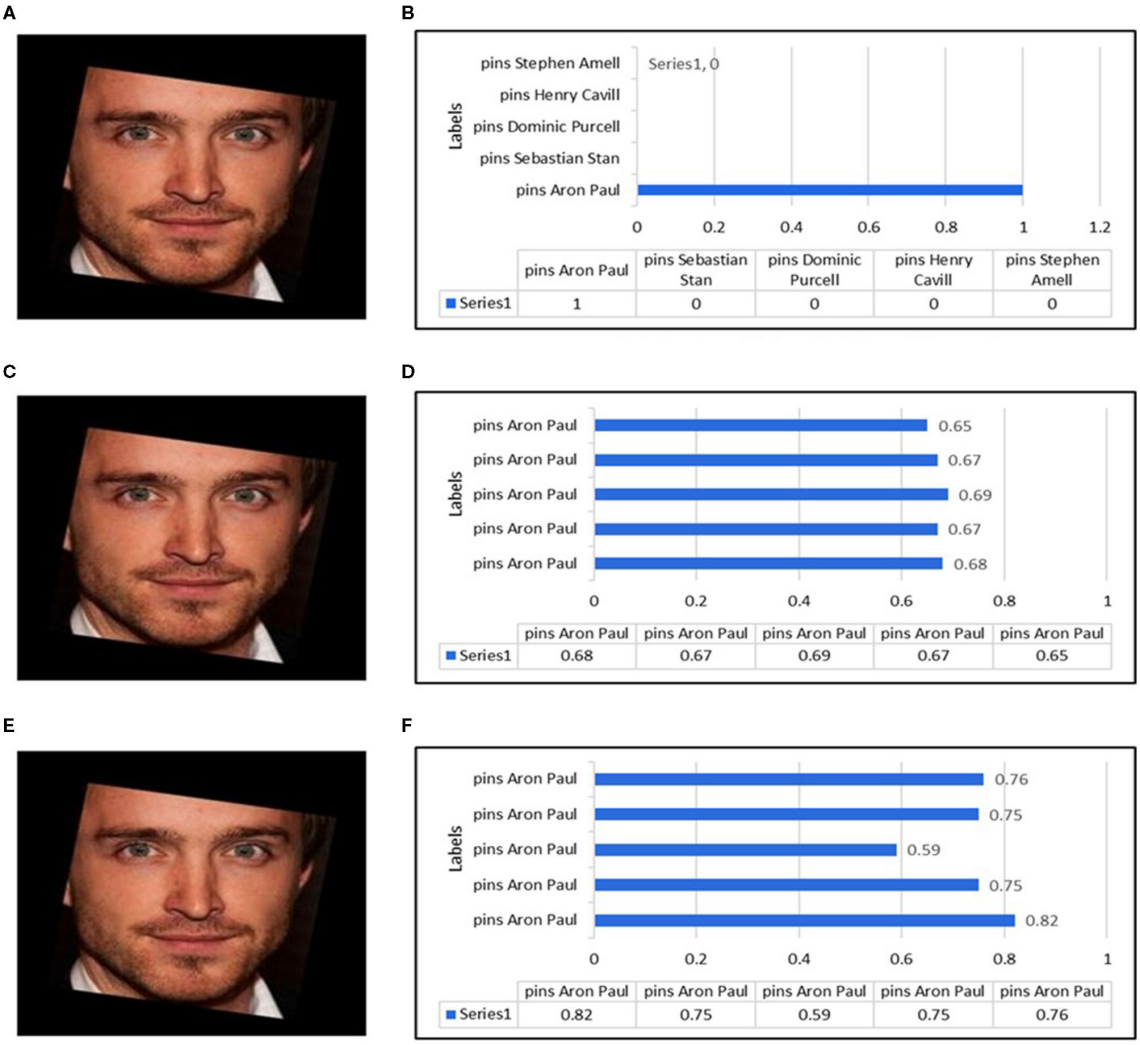
Similarity measures of most top five best found for a query. **(A)** Neural network query image. **(B)** Top five predictions by neural network. **(C)** Euclidean distance query image. **(D)** Top five predictions by Euclidean distance. **(E)** Cosine similarity query image. **(F)** Top five predictions by Cosine similarity.

To improve our methodology's results further, we employed other conventional similarity measure algorithms such as Euclidean Distance, Cosine, and k-Nearest Neighbors (KNN) for the process of image retrieval. For this process, we removed the last layer, i.e., softmax, and started to retrieve features vector against the query images. Against the retrieval of each query image, we used different similarity matrices and computed different results, as shown in Figure 6. The tabular representation of precision, recall, F1, and F2 score values against Euclidean and Cosine metrics are described in Table 1. In the case of KNN, we only displayed average precision, recall, F1, and F2 scores values in the tabular format as this algorithm only returns the belonging class where the results would be either 0% or 100%. In this context, we computed the results either the right class or the wrong one, and the final results were computed on average percentage.

**Table 1:** (%) Comparison of precision, recall, *F*1, and *F*2 scores for Aligned Face dataset.

**Matrices**	**Method**	**Precision**	**Recall**	***F*1 score**	***F*2 score**
Euclidean	Query with each feature	90.08	84.16	87.02	85.28
Cosine	Query with each feature	92.18	86.76	89.39	87.79
k-NN	Nearest Centroid (*k* = 5)	92.00	92.66	92.33	92.52

**Figure 7:**
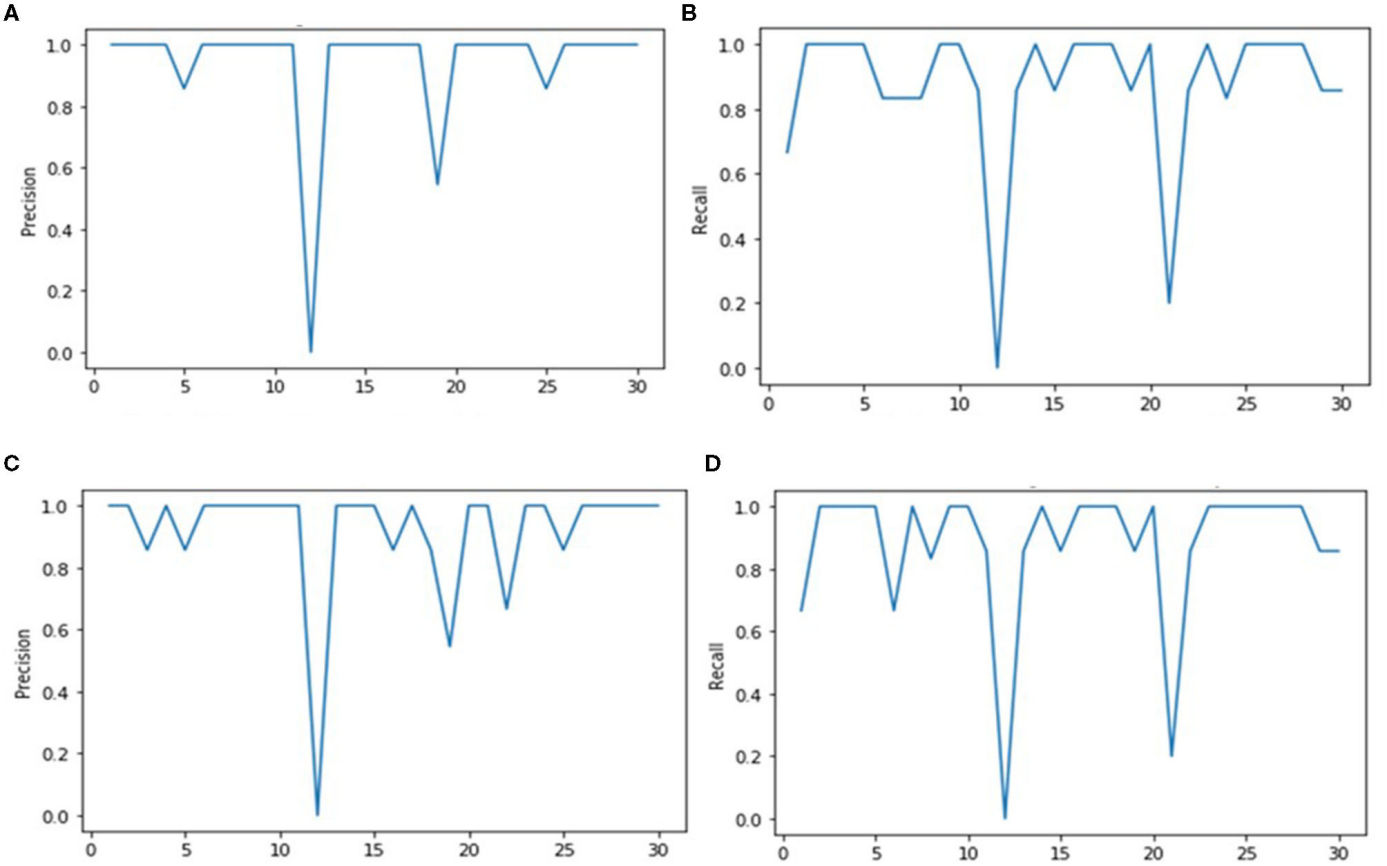
Precision and Recall scores of Euclidean and Cosine algorithms against each retrieval images. **(A)** Euclidean distance precision. **(B)** Euclidean distance recall. **(C)** Cosine precision. **(D)** Cosine recall.

**Figure 8:**
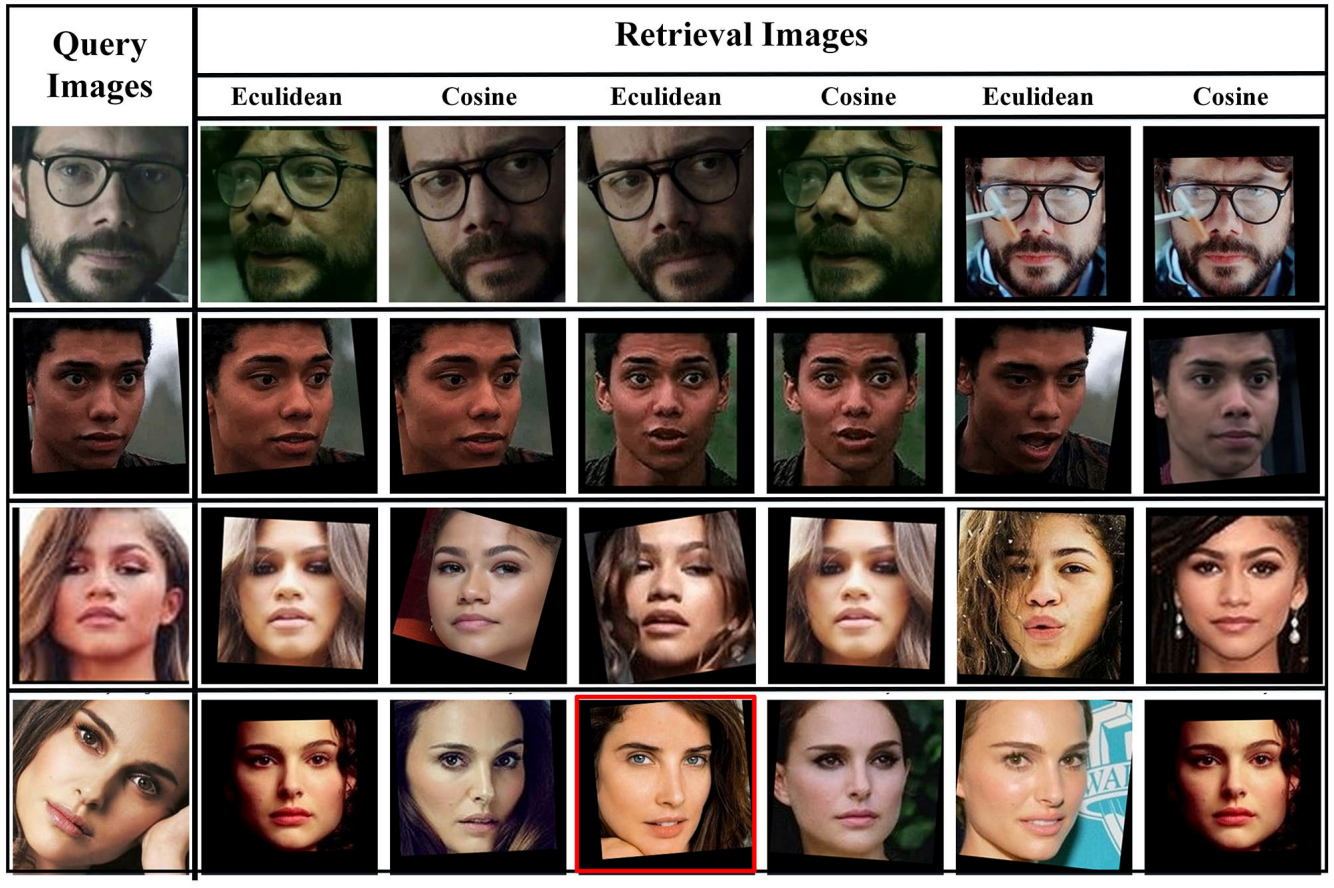
Facial image retrieval results on AFD by picking the best three images by using Euclidean and Cosine similarity scores while red box is showing wrong retrieval.

After computing results on the aligned face dataset, we used second testing holdout LFW dataset to describe our model's performance on a ratio of 4:1 for database and query images. We began again with the step of feature extraction with the help of the already trained FRAI that is well-trained on facial features/maps. After feature extraction, query images were passed to FRAI for their unique feature vector and then different similar measures, as used for the aligned face dataset, were used to retrieve images against the query. Against each query image, we calculated recall, precision, F1, and F2 score values to compute the performances as shown in Figures 7, 8, and Table 2. The results of the similarity measures are given in the following subsections.

**Table 2:** (%) comparison of precision, recall, *F*1, and *F*2 scores for LFW dataset.

**Matrices**	**Method**	**Precision**	**Recall**	***F*1 score**	***F*2 score**
Euclidean	Query with each feature	94.91	87.33	90.96	88.74
Cosine	Query with each feature	91.65	87.88	89.72	88.61
k-NN	Nearest Centroid (*k* = 5)	95.00	95.00	95.00	95.00

### 3.1 Aligned face dataset

#### 3.1.1 Neural network output

After successful training, the top five best similar classes have been computed, as shown in Figure 6B, against a face shown in Figure 6A. The training showed better results. However, the guessed class was assigned with 100% probability that it is “Aaron Paul”.

#### 3.1.2 Euclidean distance similarity

This measure showed good results with precision, recall, F1, and F2 values of 90.08, 84.16, 87.02, and 85.28%, respectively, which are relatively better than previously proposed techniques (Wu et al., 2010; Sun et al., 2019; Tarawneh et al., 2019), as shown in Table 1. The normalized threshold used for this similarity measure was 0.552 where we got the best similarity output. A threshold should be defined to assess whether the images would be retrieved or not. We assessed this threshold by the hit-and-trial method, which indicated that the images having a score of more than 0.552 are retrieved images. The graph of the top five best recall images is shown in Figure 6D for a query image in Figure 6C. Our model had shown a similar behavior with other query images.

#### 3.1.3 Cosine similarity

This measure follows the same trend as Euclidean Distance with precision, recall, F1, and F2 score values as 92.18, 86.76, 89.39, and 87.79%, respectively (shown in Table 1). It performed better at the normalized threshold of 0.72 where we got the best results. If the images have a score >0.72, it means that the database has retrieval images. The graph of the top 5 best recall images is shown in Figure 6F for a query image Figure 6E. Our model has shown similar behavior with other query images.

#### 3.1.4 k-nearest neighbors

This algorithm showed the most promising results at 92.00, 92.66, 92.33, and 92.52% for precision, recall, F1, and F2 score values, respectively, for facial-based feature vectors (shown in Table 1). The random selection method has been used for the nearest centroid classifier function with *k* = 5.

### 3.2 LFW face dataset

#### 3.2.1 Euclidean distance similarity

This measure has shown good results with maximum precision, recall, F1, and F2 score values at 94.91, 87.33, 90.96, and 88.74%, respectively, as shown in Table 2, which are comparably better than those obtained from previous techniques (Wu et al., 2010; Sun et al., 2019; Tarawneh et al., 2019). The graphs of precision and recall are shown in Figures 7A, B, respectively, for each query image.

#### 3.2.2 Cosine similarity

This measure follows the same trend as Euclidean Distance with precision, recall, F1, and F2 score values of 91.65, 87.88, 89.72, and 88.61%, respectively (Table 2). In Figures 7C, D, it is shown that the lower score peaks are higher than those from the Euclidean Distance for the precision graph, but the recall graphs exhibited a similar trend as that of the Euclidean Distance.

#### 3.2.3 k-nearest neighbors (KNN)

This algorithm showed the most promising results at 95.00, 95.00, 95.00, and 95.00% for precision, recall, F1, and F2 score values, respectively, for facial-based feature vectors (Table 2). The random selection was used for the nearest centroid classifier function with *k* = 5.

**Figure 9:**
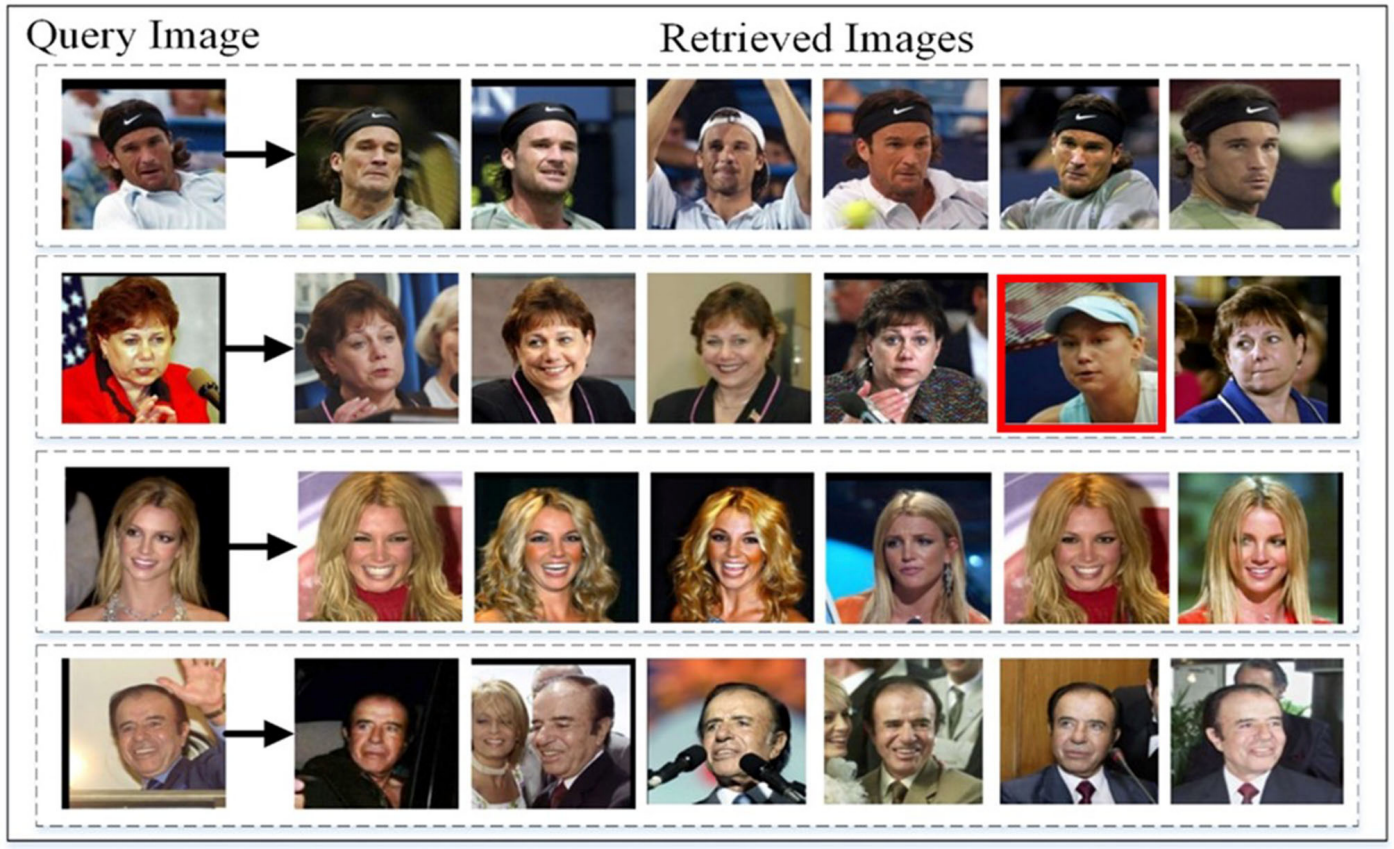
Facial image retrieval results using LFW on the best retrieved images and red box is showing wrong retrieval.

**Figure 10:**
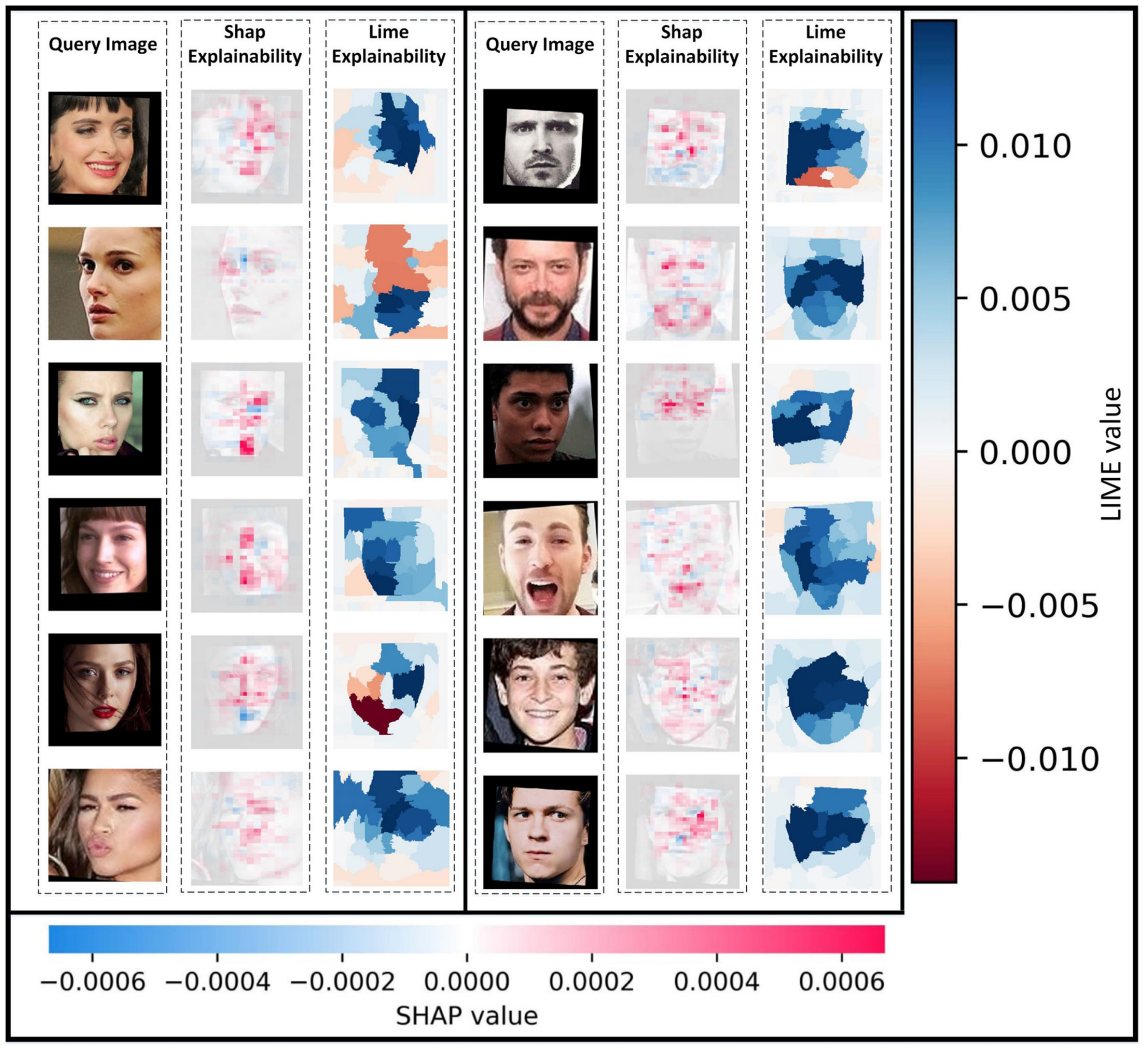
Facial landmarks' characterization based on facial expressions and appearances on AFD dataset faces by applied SHAP and LIME on the FRAI model.

The FRAI model has shown very promising results in the image recognition process for the AFD dataset irrespective of any particular facial expression. Generally, our model produces higher scores compared to those images where the same character with a similar expression exists. In the same way, our model retrieves the same appearance images for the characters with appearance particulars such as beard or glasses or any other appearance elements to better fulfill the need of a query image. Overall, these mentioned scores are not for any peculiar expression and is our model's tendency to show that expression and appearance matter in the image retrieval task, as shown in Figure 8 for the AFD dataset. Figure 10 shows the different facial expressions experienced by the FRAI model during training. Furthermore, the best-retrieved images using Euclidean measure for the test dataset have been shown in Figure 9 for the LFW dataset. The first cell in Figure 9 shows a query image. All images, following the query image, were retrieved as best images against the query image.

In comparison to the pervious techniques of Wu et al. (2010), Sun et al. (2019) and Tarawneh et al. (2019), our training model FRAI and similarity measures made our technique novel and robust. In the study of Wu et al. (2010) titled Scalable Face Image Retrieval with Identity-Based Quantization and Multi-reference Re-ranking, an average accuracy of 71% has been shown because the authors employed conventional features such as local and global features and then used these features for the retrieval of images. These authors focused on only appearance features such as eyes, nose, and ears (Wu et al., 2010) but failed to focus on the whole face map, which is a limitation in their research. On the other hand, in the study titled “Eye-tracking based relevance feedback for iterative face image retrieval” by Sun et al. (2019), the authors used first Face++ algorithm to get the top 36 ranked images, and then, they were passed to the neural network for the retrieval process and showed a good average precision recall score of 90.00% (Sun et al., 2019). This model has not shown more than 90% accuracy, which may be due to the large number (i.e., 36) of ranking images among which there may be few images that will have the similar faces/expressions but not the correct face. Similarly, the study titled “Deep Face Image Retrieval: a Comparative Study with Dictionary Learning” by Tarawneh et al. (2019) has also shown good results of 90.50% average precision recall score. In their method, they have used deep learning methods to retrieve images by analyzing the face mesh. Compared to our study, the study of these authors did not employ further any other retrieving similarity measure for the images, which is a gap in most of the methodologies and which we have also shown with the help of different metrics. All of the abovementioned three techniques have been compared with our methodology in Table 3. The comparison of previous techniques with our proposed methodology has shown a much more efficient average precision recall.

**Table 3:** Comparison with previous techniques.

**Methods**	**Average precision-recall (APR)**	**References**
Proposed methodology	95.48%	–
Deep face image retrieval: a comparative study with dictionary learning	90.50%	Tarawneh et al., 2019
Scalable face image retrieval with identity-based quantization and multi-reference re-ranking	70.50%	Wu et al., 2010
Eye-tracking based relevance feedback for iterative face image retrieval	90.00%	Sun et al., 2019

Our methodology has worked out very well due to two reasons. First, our training model FRAI is the combination of two renowned models and has shown good training and validation results. Second, we did not use it directly to retrieve images from the database. To use FRAI for feature extraction first and then against each query's feature vector, we retrieved different images with the help of different renowned similarity algorithms that have shown increased recall, precision, F1, and F2 scores. These two reasons have shown us that a good model and good feature matching algorithm, such as KNN in our case, improve the retrieval system.

In our study, we also employed two AI explainability tools, SHAP (Lundberg and Lee, 2017) and LIME (Ribeiro et al., 2016), to interpret the FRAI model results. To obtain more accurate pixel annotations and segmentations, we conducted up to 5,000 and 20,000 evaluations for each query image using SHAP and LIME, respectively. Both explainable tools were able to interpret the FRAI model in a different way. While SHAP highlights the most important pixels in the query images that the FRAI model pays attention to, LIME focuses on developing segmentations of the facial features related to the searched face image. From pixels and regions, the important segmentation aspects from SHAP and LIME, respectively, was found. With segmentation, the characters' facial expressions and appearances, such as with beard or glasses or any other visual characteristics, were helpful in retrieving, as shown in Figure 10, the related expression faces most of the time.

The use of FRAI (a tool that can perform a face recognition) together with the use of explainable AI tools (which highlight personalized facial landmarks responsible for FRAI face identification) has the potential to support protectionists to identify marker combinations and their relationships with specific face features that can be related to somatic characteristics, mood or psychological states, and pathological trajectories, among others.

Overall, our study demonstrates the potential of the FRAI model in a range of applications and highlights the value of explainable AI models in interpreting complex problems.

In conclusion, future advancements of the FRAI tool emerge as a potential decision tool in all those applications where the identification of personalized facial recognition markers may be of crucial importance, e.g., in clinical diagnostics, prognostics criminology, security, and many others.

## 4 Conclusion

In this study, we developed an efficient, more accurate, and less computational novel technique for the automation of an end-to-end facial character identification, emphasizing specifically on the extraction and then recognition of characters exhibiting diverse facial expressions. We used two standard datasets, namely the Aligned Face dataset and the LFW dataset, which consists of a number of characters posing various facial expression landmarks. We started with the Aligned Face dataset and trained our novel model named FRAI, which is a combination of GoogleNet and AlexNet, used for the feature extraction process. Then, we developed two databases against the Aligned Face dataset and holdout testing LFW dataset. For these databases, a number of metrics were used for the calculation of precision, recall, F1 and F2 scores using Euclidean distance-, Cosine distance and k-nearest neighbor (KNN) measures for the retrieval process against each query image. We achieved maximum precision, recall, F1, and F2 score values of 92.00, 92.66, 92.33, and 92.52%, respectively, for the Aligned Face dataset used for training and 95.00% for LFW dataset used for testing by using the KNN measure. Our methodology has concluded that a good facial trained model is not enough for the facial based image retrieval system. To use this model for a feature extraction process and then employ different conventional measurement algorithms for the retrieval process, it is usually recommended to increase the performance, which, in our case, is KNN.

The FRAI tool may be potentially used in the healthcare, for example, to predict different diseases from features of facial characterization such as neurological diseases and cancer, brain growth development in the cases of babies, and so on, and criminology, among other fields. In the future, we will improve our technique by developing a new database using GANs. On the newly generated faces, a new parallel and vertical combination of neural networks, namely, GoogleNet, AlexNet, ResNet (Simonyan and Zisserman, 2015), will be employed to enhance the robustness of the features toward the defined goal.

## Data availability statement

The original contributions and datasets utilized in the study are publicly accessible and have been appropriately referenced in the article/supplementary material. Further inquiries can be directed to the corresponding author/s.

## Author contributions

Material preparation, data collection, analysis, and methodology implementation were performed by STS and SAS. SQ, AD, and MD managed and guided in the development of the whole strategy. The first draft of the manuscript was written by STS and SAS, and then, all authors commented on previous versions of the manuscript. SQ, AD, and MD read and approved the final manuscript. All authors contributed to the study conception and design. All authors contributed to the article and approved the submitted version.

## Footnotes

^1^*5 Million Faces—Top 15 Free Image Datasets for Facial Recognition*. Lionbridge AI (n.d.). vailable online at: https://lionbridge.ai/datasets/5-million-faces-top-15-free-image-datasets-for-facial-recognition/ (accessed December 8, 2019).
